# Visual Cues for the Retrieval of Landmark Memories by Navigating Wood Ants

**DOI:** 10.1016/j.cub.2006.10.068

**Published:** 2007-01-23

**Authors:** Robert A. Harris, Paul Graham, Thomas S. Collett

**Affiliations:** 1 School of Biological Sciences, University of Sussex, Falmer, East Sussex BN1 9QG, United Kingdom

**Keywords:** SYSNEURO

## Abstract

**Background:**

Even on short routes, ants can be guided by multiple visual memories. We investigate here the cues controlling memory retrieval as wood ants approach a one- or two-edged landmark to collect sucrose at a point along its base. In such tasks, ants store the desired retinal position of landmark edges at several points along their route. They guide subsequent trips by retrieving the appropriate memory and moving to bring the edges in the scene toward the stored positions.

**Results:**

The apparent width of the landmark turns out to be a powerful cue for retrieving the desired retinal position of a landmark edge. Two other potential cues, the landmark's apparent height and the distance that the ant walks, have little effect on memory retrieval. A simple model encapsulates these conclusions and reproduces the ants' routes in several conditions. According to this model, the ant stores a look-up table. Each entry contains the apparent width of the landmark and the desired retinal position of vertical edges. The currently perceived width provides an index for retrieving the associated stored edge positions. The model accounts for the population behavior of ants and the idiosyncratic training routes of individual ants.

**Discussion:**

Our results imply binding between the edge of a shape and its width and, further, imply that assessing the width of a shape does not depend on the presence of any particular local feature, such as a landmark edge. This property makes the ant's retrieval and guidance system relatively robust to edge occlusions.

## Introduction

Some ants, having located a reliable source of food, shuttle back and forth along idiosyncratic, visually guided routes (e.g., 1, 2, 3, 4, 5); the final stage of their route to a goal is guided by a stored local view or snapshot of the surroundings as seen from a vantage point close to the destination (review [6]). A view of a familiar object stored as a snapshot seems to be encoded in terms of a small set of distinct features, such as oriented edges, color, and retinal size 7, 8, 9. In some experimental situations, ants and bees attend to high-contrast boundaries of objects and guide their path to their destination by moving so as to shift edges in the current retinal image toward their stored retinal positions in the snapshot 7, 9. If the visual scene is manipulated so that some edges are missing and cannot be matched to the snapshot, the insects will steer as best they can with the remaining edges 7, 9.

Routes are guided in part by a series of visual memories, which insects match sequentially 10, 11, 12. Accurate route guidance requires mechanisms for retrieving the snapshot that is appropriate for a particular point along the current route. Contextual cues such as time of day 13, 14, odor 15, 16, or motivational state 17, 18 determine which route an ant or bee takes by priming the set of memories associated with that route. Other cues, such as spatial panorama [19] and sequential position [10], act to prime particular visual memories within a route. In this paper, we examine the paths of ants, which follow a short route to collect food close to a single visual landmark, and ask what features of the landmark might prime the retrieval of a stored view of that landmark. Some features of an object seem to be particularly suitable. For instance, as the ant approaches an object, the object's image on the retina becomes larger. A measure of the object's angular size (width, height, or area) could cue the retrieval of other stored features (e.g., the desired retinal position of an edge) that may be less helpful for accessing the memory but essential for guidance.

**Figure 1:**
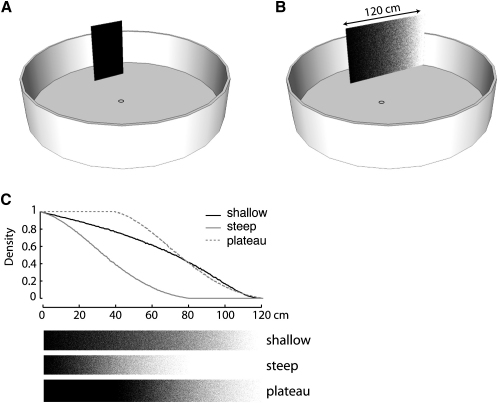
Experimental Arena and Landmarks Ants were trained with either (A) a two-edged landmark or (B) a one-edged gradient landmark. Ants were placed in a starting pot at the center of the arena. They then walked to a sucrose reward placed at the landmark's base. (C) Ratio of black to white pixels across the three experimental gradients. The slopes of the steep and plateau gradients were identical, with the plateau offset by 40 cm of solid black. The physical width of each stimulus was 120 cm. Further details are in the Experimental Procedures.

We trained ants along an 80 cm path to collect sucrose at the base of a rectangular landmark with one or two high-contrast vertical edges (Figures 1A and 1B). Sucrose was placed either at a point inset from one edge of the landmark or at the edge itself. The two-edged landmark was a black wall, and we contrived the single-edged landmark by making one vertical edge black, with the black fading along the extent of the landmark to the white of the arena background (Figures 1B and 1C).

How might ants be guided by these landmarks to approach the food? Ants trained to find food at the very edge of a gradient need only keep the edge in their frontal visual field while walking forward. The task becomes more complex with food inset from the edge. At the start of a direct approach to the goal, the edge is relatively frontal on the ant's retina but moves peripherally as the ant gets closer to the food. Our experimental findings are consistent with ants' acquiring a series of snapshot memories along their route, each containing a different desired position of the edge.

Experimental data suggest that the primary cue driving memory retrieval is the perceived angular width of the landmark, a variable that increases monotonically and reliably during an ant's approach. The angular width of the landmark and the desired position of the vertical edge are in some way linked together, so that perceived width can determine the currently desired edge position. The relatively simple situation of these experiments allows us to construct a model of snapshot retrieval and guidance. In this model, ants retrieve the appropriate snapshot and walk forward while keeping the edge in the desired retinal position (one edge) or bringing edges toward the desired position (two edges). The model generates idealized paths that can be compared with the ants' real paths.

## Results

### Paths Followed in Training Conditions

**Figure 2:**
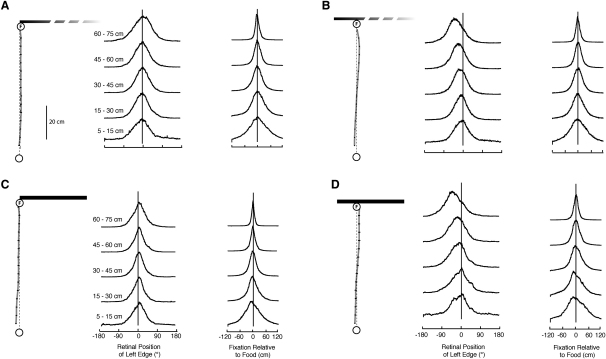
Routes of Ants during Their Approach to Training Landmarks (A–D) The left of each panel shows, for a given training condition, the mean route. Here and in the other figures, the error bars give standard errors. The columns of histograms in each panel show, for successive 15 cm bins during the approach, the retinal position of the left edge of the stimulus (left) and the ants' fixation point on the landmark (right). The histograms were calculated from every recorded frame within the 15 cm bin. In this and the following figures, N is the number of recorded ants, and n is the number of recorded paths. (A) Food at left edge of a shallow gradient (N = 22, n = 295). (B) Food 10 cm inset from left edge of a shallow gradient (N = 44, n = 838). (C) Food placed at the left edge of a 40-cm-wide wall (N = 28, n = 175). (D) Food 10 cm inset from the left edge of the wall (N = 71, n = 243).

The mean trajectories of ants in the four training conditions are shown in Figure 2. Ants follow a roughly straight path to the food, whether the food is at the edge of the landmark (Figures 2A and 2C) or inset from it (Figures 2B and 2D) and whether the landmark is a single-edged gradient (Figures 2A and 2B) or a two-edged wall (Figures 2C and 2D). The retinal positions of the left edge of the landmarks are consistent with the mean trajectories (Figure 2). With the food at the left edge of the landmark, the edge was, on average, kept in the ants' frontal visual field along the whole route. With the food inset from the edge, the edge migrated peripherally over the retina from a frontal position toward 90°. The same data plotted in terms of the ants' fixation point along the landmark show, as expected, that ants tend to fixate a point close to the goal throughout their route (Figure 2).

### Does One Snapshot Memory Suffice to Guide an Ant When It Is Trained to a Goal Inset from the Edge of a Gradient?

**Figure 3:**
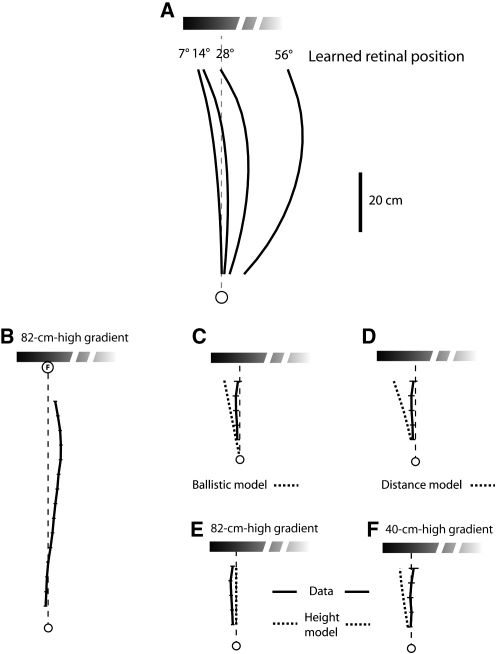
Tests for Multiple Memories and Different Retrieval Cues (A) Simulated routes generated when the left edge of a gradient is held in a single retinal position over the whole route. The fixed positions (7°–56°) correspond to single snapshots stored on the direct route at 80, 40, 19, and 7 cm from the goal. (B–F) Mean routes (solid lines) and simulated routes (dashed lines) for different landmark heights and distances. Ants were trained to an 82-cm-high left gradient placed 80 cm from the start and were subsequently tested with either an 82- or a 40-cm-high gradient placed at 30 cm from the starting location. (B) Mean route with training landmark (N = 34, n = 223). (C) Tests with the landmark 30 cm from start. The mean route combines data from tests with 40- and 82-cm-high gradients (N = 44, n = 59). The simulated route supposes that ants set their initial direction with the left edge placed 7° to the left of their midline. (D) Mean path, as in (C). Simulated path with distance from start controlling the retrieval of desired edge positions. (E and F) Mean path from tests with 82-cm-high ([E] N = 35, n = 116) or 40-cm-high ([F], N = 9, n = 43) gradient at 30 cm from the start. Paths are simulated with apparent height controlling the retrieval of desired edge positions.

Could the ant be guided to a position 10 cm from the edge of a gradient with just a single snapshot memory? If the ant took a snapshot of the gradient edge at the start of its journey and was guided by that snapshot throughout the route, it would keep the edge 7° left of its midline, and its path would curve to the left as it approached the gradient. Figure 3A shows a family of curves for different learned retinal positions of the left edge. The curvature of the paths increases with the eccentricity of the stored edge position. This strategy does not result in the approximately straight route to the goal that is observed experimentally.

A second strategy for navigating a route without multiple memories is for the ant to learn an initial direction in which to head relative to the edge of the gradient. The ant might start its journey in that visually defined direction but then continue ballistically. Evidence that ants do not normally use this strategy comes from releasing them nearer to the gradient than in training (Figure 3C). Ants that had been trained along an 80 cm route to a feeder that was inset 10 cm from a left-edge gradient (Figure 3B) were tested with the same gradient or with a gradient approximately half the height, placed at 30 cm from the start. If ants used their initial snapshot to pick a direction and then followed a ballistic strategy, the predicted direction of their paths would be to a point 7.1° right of the edge, as measured from the start; this would be equivalent to heading 11.3° to the left of the food. Instead, the mean direction in which the ants were heading at 25 cm from the start was approximately straight ahead; these bearings differ significantly from the prediction of a ballistic strategy (Figure 3C; 0.9° ± 4.3°, 99% confidence interval [CI], p < 0.01).

### Does Snapshot Retrieval Depend on Monitoring Distance Walked?

If the approach to the landmark is guided by several snapshots, what cue or cues prime their retrieval? One possibility is that the retrieval of each snapshot is linked to the distance that an ant has walked from the starting point. In this case, ants should aim incorrectly when the landmark is placed at an unexpected distance from the start. Thus, the data of Figure 3C also imply that distance from the start is not a retrieval cue. If it were, the ants' paths would veer to the left (Figure 3D) and at 25 cm from the start would be directed 15.1° to the left of the goal, whereas the ants' mean path is straight ahead (0.9° ± 4.3°, 99% CI, p < 0.01).

### Does the Apparent Height of the Landmark Drive Snapshot Retrieval?

Several parameters related to the apparent size of the landmark increase monotonically as the ant approaches the landmark, and these could provide information for retrieving a snapshot memory. One possibility, which our data exclude, is the landmark's apparent height. Consider again the tests of Figure 3, in which ants approach a left-edge gradient 30 cm from the start. Figure 3E shows the ants' mean path when the gradient was 82 cm high, as in training, to be compared with paths when the gradient was 40 cm high (Figure 3F). If apparent height primed snapshot retrieval, the paths with the 40-cm-high gradient would turn to the left, and those with the close 82-cm-high gradient would aim at the food position. The data contradict these predictions. Both sets of paths point roughly at the food position, with no significant difference in their bearings (Watson-Williams F test, df = [1, 19], p = 0.17). The mean path to the 82-cm-high gradient, as measured 25 cm from the start, was directed just to the left of the goal (2.8° ± 3.8°, 99% CI), and that to the 40 cm high gradient was directed to the right of the goal (−2.1° ± 9.6°, 99% CI); these findings differ significantly from the predicted direction of 8.1° to the left. Furthermore, in a separate experiment (data not shown), ants took straight routes when they were trained to approach food placed 10 cm from the left edge of a 40-cm-wide two-edged landmark (as in Figure 2D), with the height of the landmark varied between each training trial. Stable height is not essential for acquiring a direct route to the goal, nor is height a strong cue for retrieving snapshots.

### Does Snapshot Retrieval Depend on Apparent Landmark Width?

**Figure 4:**
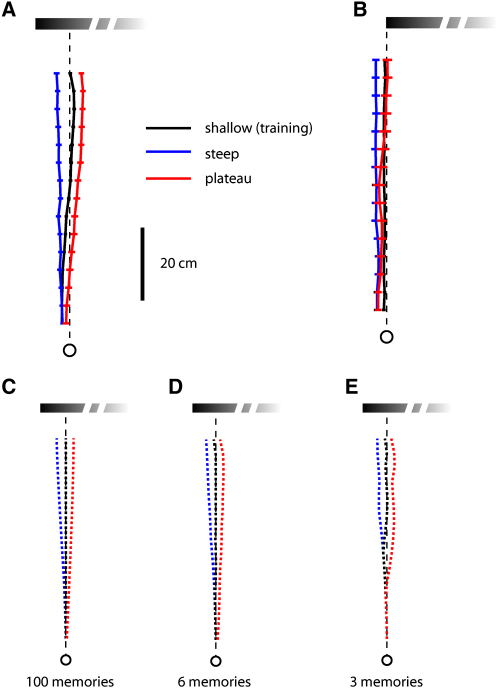
Ants Trained with the Shallow Gradient (A) Mean routes of ants trained to find food 10 cm from the left edge of the shallow gradient and tested with a narrower (steep) or wider (plateau) gradient (shallow, N = 16, n = 308; steep, N = 14, n = 70; plateau, N = 14, n = 87). (B) Mean routes of ants trained to find food at the left edge of the shallow gradient and tested with steep and plateau gradients (shallow, N = 10, n = 209; steep, N = 9, n = 52; plateau, N = 9, n = 34). (C–E) Different numbers of memories were used for simulating routes to the three gradients. Desired edge positions are retrieved according to the apparent width of the landmark.

The apparent width of the landmark also increases reliably during the ants' approach, and manipulations of landmark width have a significant effect on the ants' routes. Figure 4A shows the mean paths of ants trained to a feeder placed 10 cm in from the left edge of a shallow left-edge gradient and then tested with narrower (steep) and wider (plateau) left-edge gradients. Ants tend to walk leftward of the straight line route when presented with the steep gradient and rightward when presented with the plateau gradient (Page's L test, 11 ants, three conditions, L = 151, p < 0.001).

One possible explanation for this behavior is that the ants' paths are biased toward the center of gravity of the gradient stimuli. Weak evidence against this hypothesis comes from training ants to feed at the left edge of a shallow gradient. Ants trained this way and tested with steep and plateau gradients continued to walk in a straight line, which was unaffected by the change in the gradient's width (Figure 4B) (L = 73, six ants, three conditions, p > 0.05). Changing this visual characteristic of a landmark does not always influence an ant's route.

The data suggest instead that ants use some measure of the visual width of the landmark to retrieve the appropriate snapshot memory. According to this hypothesis, each snapshot memory contains the landmark's apparent width linked to the corresponding desired edge position. It is as though the ant stores a look-up table, each row of which contains one value of angular width and its associated desired edge position, with width providing an index for retrieving the corresponding edge position. As the ant approaches the goal, the increasing width triggers the retrieval of snapshots with the left edge at increasingly eccentric positions. In the remainder of the paper, we show that this hypothesis holds for a variety of experimental conditions.

Landmarks that are narrower or wider than the training landmark will cause ants to retrieve snapshots meant for earlier or later positions on their route, respectively. A memory retrieved from later in the route will lead the ant to place the edge of the left-edge gradient more peripherally than usual and walk to a point right of the training route. Conversely, the retrieval of a memory from earlier in the route will cause the ant to place the edge more centrally on the retina and to walk left of the training route. A model implementing this mechanism (see Experimental Procedures) generates paths that resemble the ants' mean paths in tests with the steep and plateau gradients (Figure 4C). The model generates roughly similar paths with six snapshot memories (Figure 4D), and even with only three (Figure 4E).

This model also explains why changing the width of the landmark has no systematic effect on the paths of ants trained to food at the edge of the gradient (Figure 4B). In this case, the desired edge position is at 0° for all the stored values of apparent width.

**Figure 5:**
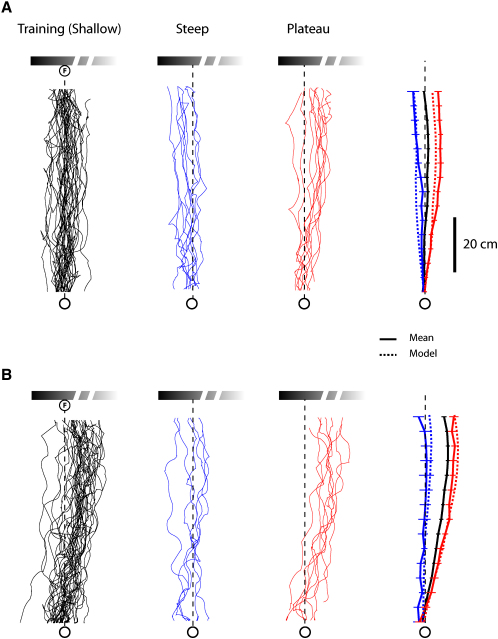
Individual Routes of Ants Trained with the Shallow Gradient (A and B) Individual, mean, and simulated paths of two ants. (A) Routes similar to the population mean (shallow, n = 35; steep, n = 8; plateau, n = 10) and (B) routes when training was consistently biased to the right of the population mean (shallow, n = 30; steep, n = 6; plateau, n = 8). Simulated paths are generated from a look-up table of memories based on the individual's mean training route.

The same model can also account for the paths of individual ants. Figure 5 shows the paths of two individuals. The routes of one ant (Figure 5A) are similar to the mean route of the recorded population (Figure 4A). Those of the second ant (Figure 5B) differ significantly from the population mean, with training routes that curve to the right. The routes of these two ants to the steep and plateau gradients differ in a manner that suggests that the ants have learned different associations between desired edge position and apparent landmark width when running their training routes and that they apply their idiosyncratic look-up tables when approaching the test gradients. Indeed, simulated paths generated this way resemble the ants' paths (Figure 5 and see Experimental Procedures).

### Approaching Landmarks with Two Vertical Edges

**Figure 6:**
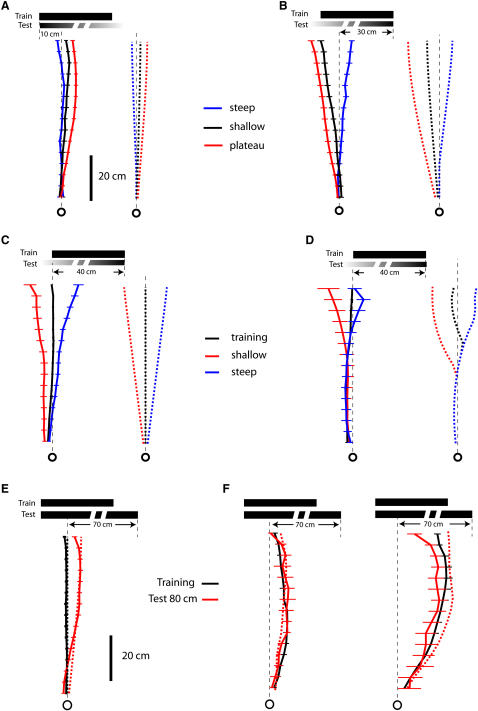
Ants Trained to Two-Edged Landmarks (A–F) Mean and simulated routes after training to a 40-cm-wide two-edged landmark. (A and B) Ants trained to food 10 cm in from the left edge. (A) Tests with left-edge gradients (steep, N = 16, n = 70; shallow, N = 16, n = 65; plateau, N = 16, n = 82). (B) Tests with right-edge gradients (steep, N = 21, n = 60; shallow, N = 15, n = 62; plateau, N = 24, n = 66). (C and D) Ants trained to food at the left edge and tested with right-edge gradients. (C) Mean routes of the population (training, N = 16, n = 143; steep, N = 17, n = 64; shallow, N = 15, n = 58). (D) Mean routes of an idiosyncratic ant (training, n = 14; steep, n = 6, shallow n = 5), with routes to right-edge gradients simulated from three snapshots. (E) Experimental and simulated paths of ants trained to a 40-cm-wide landmark and tested with an 80-cm-wide landmark (40 cm, N = 23, n = 262; 80 cm, N = 22, n = 98). (F) Mean and simulated routes of two idiosyncratic individuals (40 cm, n = 11 and 8; 80 cm, n = 5 and 4).

Does the same model work for approaches to a more complex landmark with two vertical edges, rather than one? Ants were trained to approach a feeder inset 10 cm from the edge of a 40-cm-wide, black, two-edged landmark and then tested with left and right gradients of different widths. The three left-edge gradients each cause the ants to take different routes (Figure 6A), most leftward for the steep gradient and most rightward for the plateau (L = 174, 13 ants, three conditions, p < 0.001). When tested with right-edge gradients (Figure 6B), the effect on the routes is reversed (L = 132, 10 ants, three conditions, p < 0.001). The spread of the paths generated by the right-edge gradients is significantly wider than is the spread generated by the left-edge gradients (Wilcoxon rank-sum test, p < 0.001). The spread is defined as the difference between the mean lateral positions (measured between 55 and 75 cm from the start) of the trajectories observed in the plateau- and steep-gradient tests.

In both these characteristics (spread and order), the paths agree well with the qualitative predictions of the model. During the early part of the route, the right edge of the two-edged landmark moves more rapidly to the periphery of the retina than does the left edge. Consequently, when gradient width is changed in tests and snapshots are thus retrieved earlier or later than usual, deviations from the normal route will be greater with the right- than with the left-edge gradient.

Even though the two-edged training landmark looks very different from the gradients presented in the tests, the fit to the model and the fact that the paths to the left-edge gradients (Figure 6A) are similar to those obtained from ants trained with a left-edge gradient (Figure 4A) reassure us that the ants are applying part of the snapshot information that they acquired during training. These experiments suggest that each stored snapshot of the landmark holds information about the left and right edges and that the retrieval of each snapshot is primed by the width of the gradient. If one edge is missing in the ant's current view, the ant can use part of the snapshot information and guide its journey with the remaining edge.

Ants also store the positions of both edges in their snapshot when they are trained to approach food at the left edge of a 40-cm-wide two-edged landmark, even though they could just have learned to aim at its left edge. The evidence comes from testing ants with steep and shallow right-edge gradients. If ants saw a right-edge gradient as having the same width as the training landmark, they would aim at a point on the gradient 40 cm left of the edge. However, ants seem to gauge the steep gradient as narrower than the training landmark and the shallow gradient as wider (Figures 6A and 6B). Accordingly, routes to the steep and shallow gradients are, respectively, to the right and left of the 40 cm point and differ significantly from each other (Figure 6C, Wilcoxon rank-sum test, p < 0.001), in agreement with the model.

Further evidence for storage of the position of the right edge in ants trained to the left edge of the wall are the idiosyncrasies of some individuals (Figure 6D). Two ants tested with either the steep or the shallow right-edge gradients aimed at a position 40 cm to the left of the edge for more than half the route, consistent with moving in a direction set by the initial memory of the right edge. Figure 6D shows the mean routes of one of these ants. The model generates similar routes if the look-up table is limited to three snapshots (Figure 6D).

### Idiosyncratic Routes with Two-Edged Landmarks

The look-up-table model of memory retrieval can also explain the unexpected behavior of some ants that were trained to approach sucrose placed 10 cm from the left edge of a 40-cm-wide two-edged landmark. The mean population route in tests with an 80-cm-wide two-edged landmark was, as the model predicted, significantly to the right of the training route (Figure 6E, Wilcoxon rank-sum test, p < 0.005).

Some ants had an unusual training route that arced strongly to the right (Figure 6F). Their paths with the 80-cm-wide two-edged landmark were almost the same as their training routes. A model using an atypical look-up table acquired along the ant's atypical training route generates similar test routes. Why do ant and model treat landmarks double the width as though they were the training landmarks? With a typical straight route, as the apparent width grows during the ant's approach to the sucrose, the desired retinal position of the left edge shifts peripherally from 7° to 90°. But, in this case, the ant's training route first curves to the right and then back to the center so that the desired edge position starts by moving peripherally but then returns to the center. When the ant or the model applies this atypical look-up table to the 80 cm landmark, it begins by curving to the right, although less steeply than with the training landmark, and then curves back to the left. This unexpected fit between data and model is strong support for the hypothesis that ants do indeed store apparent width as an index for accessing desired edge position and use their memories of edge position to guide their route.

## Discussion

The routes of the ants in these experiments are consistent with the following model. An ant's approach to a point at the base of the landmark is guided by several snapshots, each taken at a different distance from the goal. Each snapshot includes the vertical edges of the landmark (one or two, depending on the landmark) and some measure of the landmark's apparent width. Ants retrieve the snapshot that most closely matches the landmark's apparent width and guide their path by walking in a direction that moves the landmark's vertical edges to the position dictated by the current snapshot. This model explains the population behavior of ants, and more impressively, it also explains the unusual behavior of individual ants with idiosyncratic training routes (Figures 5, 6D, and 6F and Supplemental Data available online). The wide range of routes that can be accommodated by the model gives us confidence that, to some degree, it encapsulates what the ant has stored and how it retrieves the information.

Our current findings, considered alone, give us no good reason for preferring a sequence of discrete memories, as implied by the look-up table analogy, over a more continuous representation. The reason for describing the model in terms of discrete memories comes from previous work [9]. The primary purpose of the model presented here is to emphasize the link that the ant forms between apparent width, acting as a recognition cue, and desired edge position, acting as a guidance cue.

In its current form, the model will fail if apparent width does not increase monotonically during an ant's approach. For example, if the goal is to the left of the left-hand edge, apparent width first increases as the ant approaches the goal but then decreases as the landmark is viewed increasingly eccentrically. In consequence, the index to the look-up table is ambiguous—the same visual width is associated with two different snapshots appropriate for different stages of the journey. For the model to work in such a situation, it would need to incorporate additional retrieval information—for instance, the order in which snapshots should be retrieved. The model presented here also ignores several other factors known to be important in visual route guidance; examples include compass information (e.g., 20, 21), inertia [22], and motor learning 23, 21, 24.

We know nothing about how the angular width of a landmark is measured, though other work shows that angular size is important for landmark guidance in ants 25, 26. Our data also say nothing about where width is measured (e.g., along a narrow horizontal strip of the landmark or an average taken over the whole height of the landmark). It is also unclear whether apparent width is encoded explicitly as a value in the snapshot or whether it is computed as the difference between the retinal positions of the two edges or, in the case of a gradient, as the difference between the center of gravity 27, 28 of the gradient and its edge. But two points about width are worth emphasizing. First, width can be estimated from intensity profiles that vary greatly between training (e.g., a two-edged wall) and test conditions (e.g., a gradient). Second, landmark width can be gauged and used as a retrieval cue when the landmark is not in its stored retinal position (e.g., [29]).

In a limited sense, ants approaching the landmark are recognizing it as an object. They approach a specific point on the landmark by employing a sequence of stored views of the landmark. Each stored view comprises a collection of features, of which the most important for controlling the ant's approach are the object's vertical edges. Shape-discrimination studies in honeybees 30, 31 and *Drosophila* 28, 32 indicate that shapes are encoded in terms of a limited set of visual features. There is increasing evidence for binding or links between visual features comprising a snapshot 33, 34, 35, 31. This binding aids reliable shape recognition. The current experiments add that local features not only are linked to each other but may also be linked to globally calculated shape properties, such as its apparent width or center of gravity, that do not rely on the presence of any single local feature. For instance, if apparent width were measured in terms of the angular separation between two specific edges, the computation would fail if one edge became occluded or was replaced by a gradient. The use of such global parameters will increase the robustness of shape recognition when, for instance, shadows or other visual noise eliminates some local features.

In addition to the binding of features within snapshots, there is also evidence that bees following a route link separate snapshots together 36, 11. The binding of snapshots can be considered similar to the encoding of an object. In some simple models of primate shape and object recognition, a local view of an object is encoded as a bound collection of features and an object is encoded as a collection of views from different vantage points 37, 38.

## Experimental Procedures

### Experimental Arrangements

Experiments on laboratory colonies of wood ants (*Formica rufa* L.) were performed in a circular white arena (50 cm high and approximately 2 m in diameter) (Figure 1). The arena lay within a larger experimental area surrounded by white curtains and floored with roughened sheets of white plastic. In later experiments, the area over which the ants walked was covered with one or two sheets of A0 (118 cm by 84 cm) white paper. During training, ants were placed in a cylindrical starting pot at the center of the arena. The starting pot (3 cm high, 7 cm diameter) had a single 1 cm slit through which the ants exited. To ensure that the only consistent cue to the location of the sugar was the experimental landmark, landmark and reward were rotated within the arena between every training trial. This procedure reduced the likelihood that odour cues would play a role in guidance. We also cleaned the plastic floor with ethanol or, when using paper, changed the paper regularly, and between each trial we shifted the paper relative to the landmark. The sucrose feeder at the base of the landmark was a shortened microscope slide with a centered well containing 30% w/v sucrose solution.

### Landmarks

Two types of landmark were used in training. The first, with two vertical edges, was a rectangle, 40 cm wide and 82 cm high, faced with matt black paper. The second landmark was a “gradient,” the same height as the two-edged landmark but wider (120 cm), providing a single black-white edge (Figure 1B). Gradients fading from black to white were printed with a large-format inkjet printer. Each gradient was generated from a grid of 0.5 mm by 0.5 mm pixels. A probability density function determined whether a given pixel should be printed as black or white, with the probability varying across the width of the landmark. To minimize contrast differences between the white edge of the gradient and the background of the arena, we attached a white paper flange to the edge of the gradient and aligned it flush with the arena wall. The same paper stock was used for gradient, flange, and arena wall.

### Testing

Because of the rotation of the landmark between training trials, ants were slow to learn consistent routes. In order to lessen the variability of the recorded trajectories, we waited until the ants had completed 70 training trials before introducing test conditions. No food was present during tests. The ants' routes were recorded individually with a computer-controlled video-tracking system 39, 40 that gave the ant's position and body orientation at 50 Hz. For comparison with the test conditions, we recorded a training trial before each test.

### Data Analysis

Routes were analyzed with software written in Matlab (The Mathworks). Some paths were excluded from analysis, either because the ant did not reach the landmark within 6 min or because the paths were outliers, defined as those that strayed more than 40 cm left or right of the trained route or were more than 10 times longer than the trained route (i.e., 8 m). In different experiments, the ratio of outliers to recorded routes ranged between 2.8% and 24.1%, with only two experimental conditions being above 15% (Figure 6C). We calculated individual ants' mean routes (normalized to the position of the landmark) by binning the ants' position in successive 5 cm bins along the line connecting the start and feeder. The retinal positions of the edges of the landmark (Figure 2) were computed from the ant's position and body orientation relative to the landmark, under the assumption that the ant's head is usually aligned with its body axis—an assumption supported by high-magnification video recordings [41].

We used Page's L test for ordered alternatives [42] to test the statistical significance of differences between routes generated in three or more experimental conditions. This test compares the observed rank order of the routes of each individual with a predicted ranking (provided in each case by the model predictions). This statistical method is useful when interindividual variation is large, and it also avoids pseudoreplication. We included all ants from which we collected three or more trials in each experimental condition. The rank order of the individual's routes across the experimental conditions was given by the mean signed distance of the ant from the line connecting start and food locations while the ant was between 30 and 50 cm from the start. For cases in which routes were compared between two experimental conditions, we applied a Wilcoxon rank-sum test to data assembled in the same way [42]. For the data of Figure 3, we used the angular mean and confidence intervals to decide whether the observed mean angle differed significantly from the predictions [43], and we used the Watson-Williams test to determine whether the mean angles of two samples were significantly different [43].

### Modelling

All simulations were performed in Matlab. The model simulates an ant walking from a fixed starting position to a point at the base of a two-edged or gradient landmark. To simulate memories acquired in training, we calculated a look-up table that contained the desired retinal position of the landmark edge or edges (assuming that the ant faces the food site), along with angular width of the landmark at 100 locations, equally spaced between the start and goal. The value of each stored width provides an index for retrieving the associated desired position of the landmark edges.

At each step during simulated tests, the model determines the apparent retinal width of the test landmark, finds the nearest matching entry in the look-up table (either smaller or larger), and retrieves the desired retinal edge position(s). The simulated ant then rotates to place the landmark in that desired position, moves forward 0.1 cm in that direction, and repeats the process.

The apparent perceived width of gradient stimuli cannot be calculated directly because we do not know how their intensity profiles are encoded by the visual system. Instead, we determined equivalent widths of two-edged landmarks to substitute for the gradient based on a coarse fit to the experimental data shown in Figures 6A and 6B. In this data set, the observed routes to the shallow gradient are best fit if one assumes the gradient has an equivalent width of 50 cm (to the nearest 10 cm), and those to the steep gradient are best fit if one assumes a width of 30 cm. We assumed a width of 70 cm for the plateau gradient because it has the same intensity fall-off as the steep gradient (Figure 1C), but with 40 cm of black added to one side. These widths computed for the data of Figures 6A and 6B were then applied to all of the remaining simulations involving gradient stimuli (Figures 4, 5, 6C, and 6D). A similar training look-up table generated predicted routes when distance walked or apparent visual height was tested as a possible retrieval cue (Figures 3C–3F). The route memories of an individual ant were simulated by a look-up table containing the edge positions and width measured along that individual's mean training route. Its simulated test paths were then generated from its look-up table. Further analysis of the fit of the model to the data is given in the Supplemental Data.

## Supplemental Data

Document S1. Additional Discussion, Two Figures, and One Table
