# Supplementary material for: Visual Cues for the Retrieval of Landmark Memories by Navigating Wood Ants
Source: Curr Biol. 2007 Jan 23;17(2):93–102. doi: 10.1016/j.cub.2006.10.068 (PMC1885948; doi:10.1016/j.cub.2006.10.068)
Supplement: Document S1. Additional Discussion, Two Figures, and One Table [file mmc1.pdf]

Supplemental Data

Visual Cues for the Retrieval of Landmark Memories by Navigating Wood Ants

Robert A. Harris, Paul Graham, and Thomas S. Collett

Supplemental Discussion

Idiosyncratic Paths

We show here more paths of individual ants. Ants that are trained to find food at the edge of a shallow gradient exhibit striking individual differences in their paths (Figure S1). The paths of some ants resembled the mean path and went directly to the edge. Other ants went consistently to the left of the edge, and others went to the right.

Interesting differences in the routes acquired by individual ants were also found when ants were trained to approach a two-edged landmark that varied continuously in width. From trial to trial, landmark width was varied randomly between 20 and 80 cm, with the food inset 10 cm from the left edge on all trials. We recorded routes for 20, 40, and 80 cm landmarks (Figure S2). Ants trained under this regime could perhaps have used the ratio of height to width to categorize the training widths and acquire a direct route to the feeder for different width categories. This outcome was not observed. Instead, the mean routes (Figure S2A) differed between the three widths (L = 107, 8 ants, 3 conditions, $p < 0.01$), as though the ants had acquired a single look-up table, which they applied to landmarks of all widths. On average the routes were most direct when ants approached the 40-cm-wide landmark. However, there were marked individual differences. One ant approached the feeder most directly when the landmark was 40 cm wide (Figure S2B), with paths resembling the population mean, and another when it was 20 cm wide (Figure S2C). The two ants may have acquired sets of snapshots for different training landmarks. We simulated the behavior of the two ants with look-up tables based on their paths to the 40 cm landmark and then generated routes to the 20- and 80-cm-wide landmarks. The simulated routes correspond reasonably well with those of the individual ants.

How Well Do the Model and Data Match?

We have already shown that the test data match the model qualitatively in that the model predicts the relative bearings and the rough magnitudes of the ants' differing approaches to narrow and wide walls and gradients. Here we attempt to see how well the simulated routes match the observed routes. The paths of different individuals differ in a consistent manner from individual to individual, but the paths of each individual also differ between runs (Figures S1 and S2). Thus, it seems appropriate to look for fits between the model and data at the level of the mean routes of individual ants.

Our procedure was to restrict analysis to individuals from which we had a reasonably large data sample (five or more training runs and four or more test runs) and then for each individual determine a look-up table

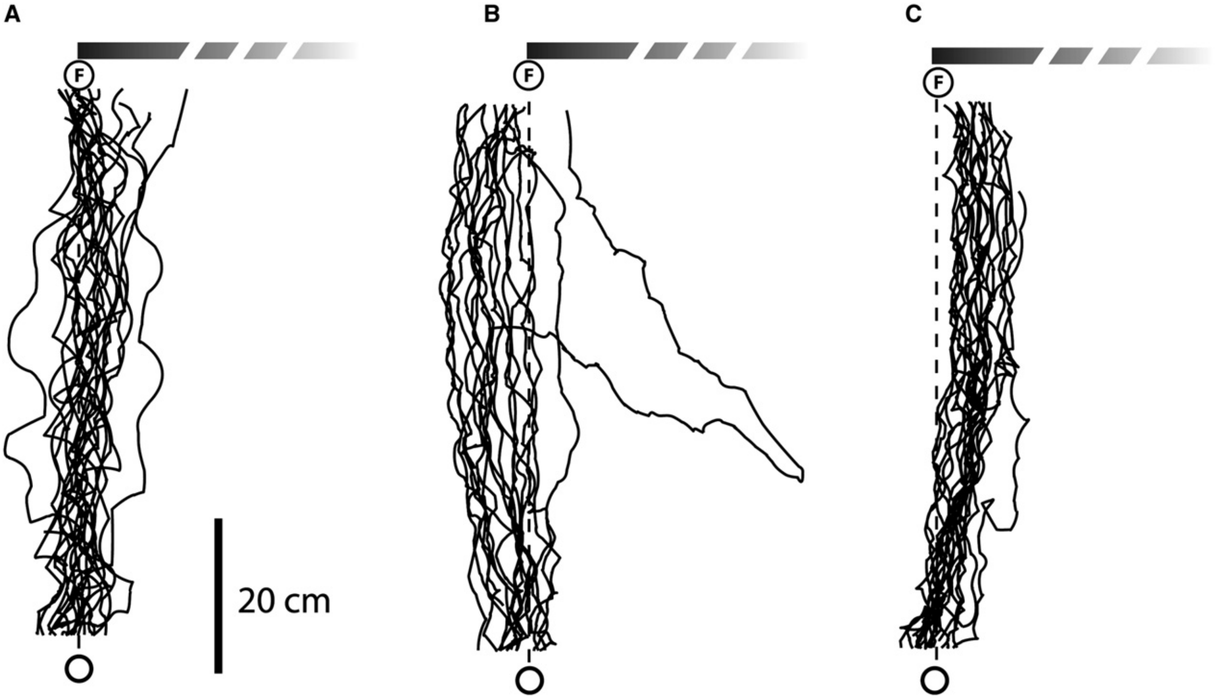

Figure S1. Idiosyncratic Routes of Individual Ants

Routes from three ants trained to find food at the edge of the shallow gradient, as in Figure 2A in the main text. In (A), the ant walked straight to the food, n = 18. In (B), (n = 15) and (C) (n = 16), the ants walked consistently to a point to the left or right of the food.

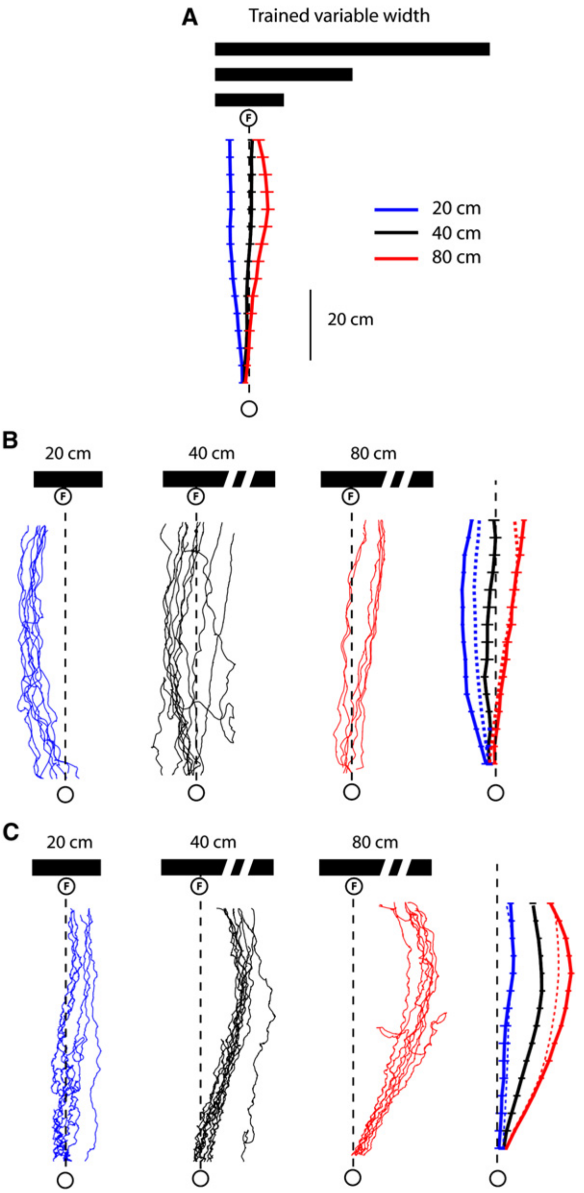

Figure S2. Routes of Individual Ants Trained with a Two-Edged Landmark of Varying Width

(A) Mean routes of ants trained 10 cm from the left edge of a landmark that varied in width between 20 and 80 cm. Routes were recorded with 20-, 40-, and 80-cm-wide landmarks (20 cm, N = 10, n = 56; 40 cm, N = 11, n = 64; 80 cm, N = 9, n = 47).

(B and C) Individual, mean, and simulated paths for two ants (20 cm, n = 8 and 6; 40 cm, n = 10 and 10; 80 cm, n = 6 and 7).

from its mean training path. The individual's paths to the test landmarks were then simulated from this look-up table; these paths provided the spine of a corridor that varied in width between 2.5 and 20 cm either side of the spine. We scored whether 75% or more of the individual's mean test path lay within the model corridor. Table S1 shows the proportion of ants that met this criterion for the different corridor widths. In interpreting the table, bear in mind that when the training runs are variable and the sample small, the mean may not be a good representation of the ants' behavior.

Table S1. Proportion of Ants Whose Mean Test Path Fell within the Model Corridor

Corridor Half-Width (cm)	2.5	5	10	20
Training: 10 cm in, Left-Edged Shallow Gradient (Figure 4A)				
Left edge steep (N = 8)	0.63	0.88	1	1
Left edge plateau (N = 9)	0.67	1	1	1
Training: 10 cm in, 40-cm-Wide Two-Edged Landmark (Figures 6A and 6B)				
Left edge steep (N = 9)	0.11	0.63	1	1
Left edge shallow (N = 8)	0.13	1	1	1
Left edge plateau (N = 12)	0.25	0.67	1	1
Right edge steep (N = 2)	0	0.5	1	1
Right edge shallow (N = 7)	0.23	0.71	1	1
Right edge plateau (N = 1)	-	-	-	-
Training: Left Edge of 40-cm-Wide Two-Edged Landmark (Figure 6C)				
Right edge steep (N = 6)	0.33	1	1	1
Right edge shallow (N = 8)	0	0.38	0.88	1
Training: 10 cm in, 40-cm-Wide Two-Edged Landmark (Figure 6E)				
80-cm-wide two-edged landmark (N = 13)	0.15	0.62	1	1
